# Amsterdam urban water system as entry point of river plastic pollution

**DOI:** 10.1007/s11356-023-26566-5

**Published:** 2023-05-16

**Authors:** Paolo Tasseron, Finn Begemann, Nonna Joosse, Martine van der Ploeg, Joppe van Driel, Tim van Emmerik

**Affiliations:** 1 Hydrology and Quantitative Water Management Group, Wageningen University and Research, 6709 PB Wageningen, The Netherlands; 2 Amsterdam Institute for Advanced Metropolitan Solutions, 1018 JA Amsterdam, The Netherlands

**Keywords:** Macrolitter, Plastic soup, Hydrology, Floating litter, Macroplastic

## Abstract

Accumulation of plastic litter in aquatic environments negatively impacts ecosystems and human livelihood. Urban areas are assumed to be the main source of plastic pollution in these environments because of high anthropogenic activity. Yet, the drivers of plastic emissions, abundance, and retention within these systems and subsequent transport to river systems are poorly understood. In this study, we demonstrate that urban water systems function as major contributors to river plastic pollution, and explore the potential driving factors contributing to the transport dynamics. Monthly visual counting of floating litter at six outlets of the Amsterdam water system results in an estimated 2.7 million items entering the closely connected IJ river annually, ranking it among the most polluting systems measured in the Netherlands and Europe. Subsequent analyses of environmental drivers (including rainfall, sunlight, wind speed, and tidal regimes) and litter flux showed very weak and insignificant correlations (*r* = $-$0.19–0.16), implying additional investigation of potential drivers is required. High-frequency observations at various locations within the urban water system and advanced monitoring using novel technologies could be explored to harmonize and automate monitoring. Once litter type and abundance are well-defined with a clear origin, communication of the results with local communities and stakeholders could help co-develop solutions and stimulate behavioral change geared to reduce plastic pollution in urban environments.

## Introduction

Plastic pollution in aquatic environments is of increasing concern because of its negative impacts on freshwater ecosystems, marine fauna, and local economies. Accumulation of plastic in urban and riverine water systems could lead to direct damage to essential infrastructure, limit water supply, and cause increased flood risks (Barboza et al. 2019; Honingh et al. 2020; van Emmerik and Schwarz 2020). It is estimated that 19–23 million metric tonnes of macroplastic enter aquatic ecosystems annually (Borrelle et al. 2020; Meijer et al. 2021). Urban water systems are assumed to be one of the largest sources of this macroplastic pollution (Tramoy et al. 2022; Van Emmerik et al. 2019), yet the relation to river plastic pollution and the connection between urban and natural water systems are poorly understood. High anthropogenic activity including recreation, open-air markets, and tourism (McCormick and Hoellein 2016; Cordova and Nurhati 2019) are assumed to be the main causes for macrolitter leakage in urban water systems. Subsequent transport to riverine and marine environments is facilitated by (extreme) rainfall events and stormwater overflow (Axelsson and van Sebille 2017), hydrologic conditions (van Emmerik et al. 2022), and other environmental factors (e.g., wind speed and wind direction) (Roebroek et al. 2021). However, a lack of observational data prevents further exploration of the abundance, transport, and retention dynamics in urban water systems.

Recent studies of plastic pollution in urban water systems aim to quantify its abundance and identify accumulation zones or hotspots. For instance, Tramoy et al. (2020) used GPS trackers in the Seine River, identifying several hotspots of plastic accumulation and observing increased floating plastic item discharges. Tramoy et al. (2022) explore the use of screened materials collected by gray infrastructures in a small urban river to characterize the macroplastic composition and mass flow. Naidoo et al. (2015) showed urban harbors to have high input and retention of macroplastics, as well as an attenuating plastic abundance further away from urban city centers. Another study by Treilles et al. (2021) examined micro- and macrolitter concentrations of suburban stormwater runoff, aiming to estimate plastic mass fluxes per hectare of urban impervious surfaces and per capita. Even though accurate estimates of urban macroplastic abundance and its spatial distribution are made, the drivers of transport and the relation to river plastic pollution are understood inadequately. Improving this understanding is critical, since many rivers are directly connected to urban water systems, which are often seen as main input locations for plastic litter (e.g., Rotterdam (Rhine) van Emmerik et al. (2022), Ho Chi Minh City (Saigon) Lahens et al. (2018), Venice (Canal Grande) Bonanno et al. (2019), Jakarta (Ciliwung) Nizardo et al. (2021), Kuala Lumpur (Klang) Zaki et al. (2021)) (Lebreton and Andrady 2019; Meijer et al. 2021)).

This paper studies the emissions of floating litter from urban water systems and its relation to riverine plastic pollution in the water system of Amsterdam. This system is characterized by a dense network of urban canals directly connected to the IJ river, from which 42 metric tonnes of floating plastic is removed annually (Waternet 2019). The IJ river is in turn flowing into the North sea, which makes the urban water system of Amsterdam relevant to study urban-natural water system connections. By conducting monthly visual counting measurements from bridges close to outlets into the IJ river, we estimated the litter outflow for Amsterdam. Subsequent comparisons with litter abundance in larger river systems were made to show urban water systems as entry points for river plastic pollution. Furthermore, correlations between potential environmental drivers of litter transport and observed litter fluxes were determined to understand their influence on emissions to the IJ river. The goal of this study is to assess and quantify the role of urban water systems as a source of river plastic pollution.

## Methods

### Study area

In this study, floating litter items were counted from bridges in Amsterdam, the Netherlands (52.373, 4.896). Home to 820,000 inhabitants, it is the largest city in the Netherlands, welcoming approximately eighteen million tourists every year (Dai et al. 2019). The urban water system in Amsterdam is characterized by extensive canals exceeding 100 km in length, consisting of multiple rings surrounding the historic and touristic city center (Pelsma et al. 2022). Northwest of the city center, the IJ river splits the urban area of Amsterdam and flows through the Noordzeekanaal to the North Sea.

**Fig. 1:**
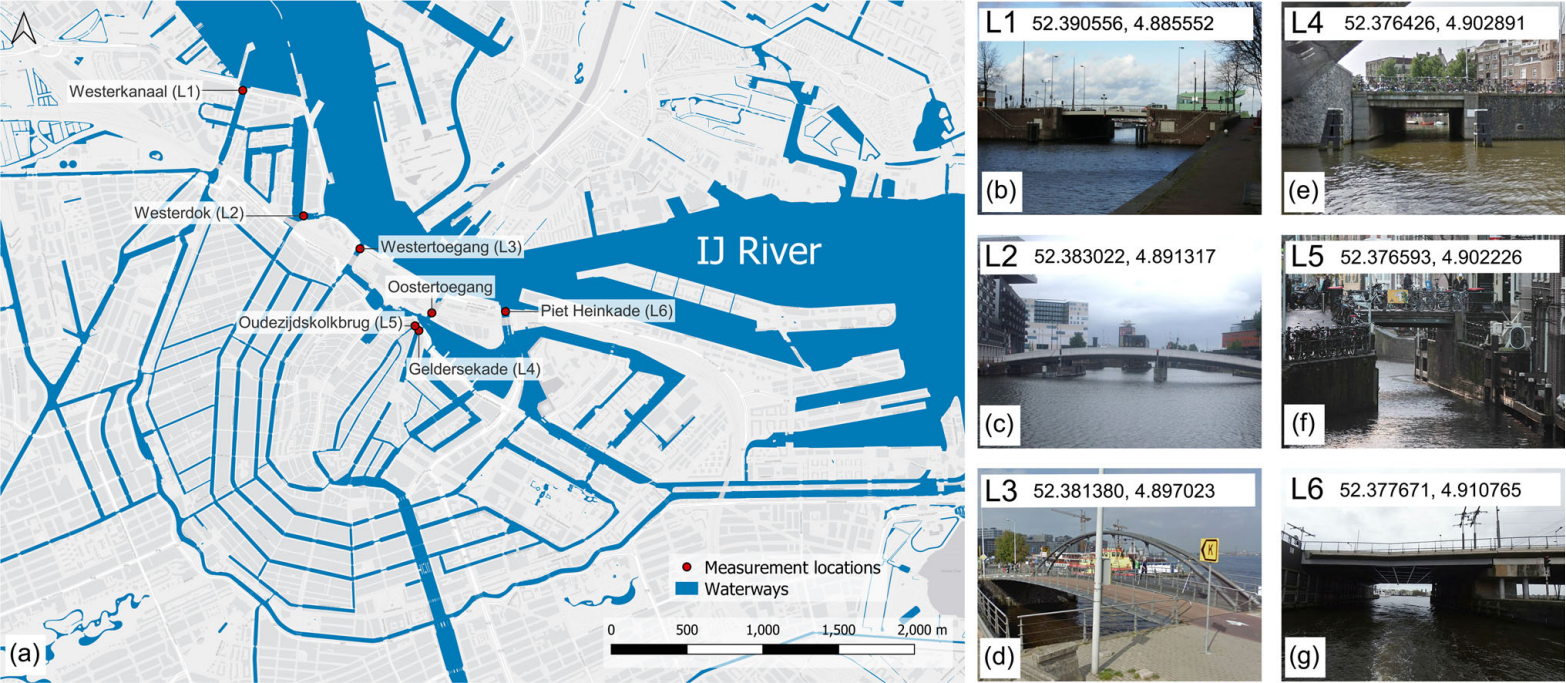
Overview of the measurement locations with **a** map including the IJ River, innercity waterways and measurement locations (bridges), and photos of **b** Westerkanaal (L1), **c** Westerdok (L2), **d** Westertoegang (L3), **e** Geldersekade (L4), **f** Oudezijdskolkbrug (L5), and **g** Piet Heinkade (L6)

The bridges used as observation locations were selected at the outlets of the six main canals in the inner city area of Amsterdam (Fig. 1). From downstream to upstream in relation to the flow direction of the IJ river, these are as follows: Westerkanaal (L1), Westerdok (L2), Westertoegang (L3), Geldersekade (L4), Oudezijdskolkbrug (L5), and Piet Heinkade (L6). Each bridge is divided into 1 to 3 segments, depending on the length of the bridge. Consequently, each segment covers a part of the canal within the field of view of the observer, enabling the identification of all floating items within a given segment.

Some bridges contain unique properties that might influence the accuracy of the results. Downstream of the Westerdok (L2), a bubble barrier (https://thegreatbubblebarrier.com/) infrastructure is installed, aiming to prevent (plastic) litter from being discharged into the IJ river. It generates a screen of bubbles, directing suspended and floating litter to a catchment system. In addition, both Geldersekade (L4) and Oudezijdskolkbrug (L5) are not situated directly at the outlet of the canal into the IJ river. Yet, since the bridge at Oostertoegang (Fig. 1) was under construction at the time of measurements, the combination of these two bridges approaches the closest estimate for litter emitted into the IJ river.

### Data collection and processing

**Fig. 2:**
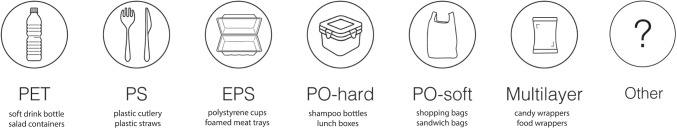
Categories used for visual counting in this research, with examples of characteristic items for these classes. The “Other” category contains all anthropogenic litter outside of the six polymer-based classes. Adopted from Tasseron et al. (2020)

Data collection was pursued through the visual counting method developed by González-Fernández and Hanke (2017). This method allows for accurate and reliable quantitative data collection of floating litter fluxes. The observer counts macrolitter items (>5 mm) in seven categories for a predetermined time interval and observation width on top of a bridge (van Emmerik et al. 2020). Based on different polymer configurations, these categories are as follows: PET (polyethylene terephthalate), PS (polystyrene), EPS (expanded polystyrene), PO Hard (polyolefins), PO Soft (polyolefins), Multilayer (multilayer plastics), and one category containing all other anthropogenic litter items (Other). The polymer types were derived based on common uses of the observed items, based on a translation table presented by van Emmerik et al. (2022). The observation detection limit of floating items depends on the bridge height (0.9$-$5.1 ms) but was estimated to be at least 2.5 cm for all observation locations. Several examples of each material category are summarized in Fig. 2, adopted from Tasseron et al. (2020).

Measurements were done bi-weekly from February 2021 until February 2022, spread over all days of the week (except Saturdays) between 7:00 AM and 7:00 PM. All observed items were logged with timestamps, location (latitude and longitude), and measurement interval duration. In total, 28 measurement days took place, with a total observed time of 37 h and 5 min. Depending on the flow velocity of the water and the level of pollution in the water system, measurements were done over a period ranging from 5 to 20 min per segment. Stationary floating items close to the bridge were not counted as discharged items and were noted in the comments of the data sheet.

The floating litter flux $F_{outlet}$ for each outlet was calculated using the following formula, adapted from van Emmerik et al. (2022):

1 $$\begin{aligned} F_{outlet} = \sum _{i=1}^{S} \frac{\bar{f_i}}{w_i} \frac{1}{S}*W*T \end{aligned}$$

in which $\bar{f}$ is the mean litter flux (items h^-1^) for bridge segment *i*, with total segments *S*, segment observation width ${w_i}$ (m), total waterway width *W* (m), and extrapolation period *T* (e.g., day, month, year). The waterway width was determined using georeferenced satellite imagery. To compute the total emission fluxes in the IJ river, the $F_{outlet}$ values of all six outlets were summed and extrapolated to a period of 1 year. In addition, an estimate of the floating litter mass transport $M_{outlet}$ was made using the $F_{outlet}$ flux, and both the mean and median mass statistics of a detailed dataset containing over 16,000 weighed macrolitter items collected from Dutch riverbanks (de Lange et al. 2023). The following equation was used to calculate the litter mass transport per outlet:

2 $$\begin{aligned} M_{outlet} = \sum _{c=1}^{7} F_c * \bar{m_c} \end{aligned}$$

in which $\bar{m_c}$ is the mean or median mass of litter category *c* (kg) (Fig. 2), and $\bar{F_c}$ the mean litter flux of the associated category (items h^-1^).

**Fig. 3:**
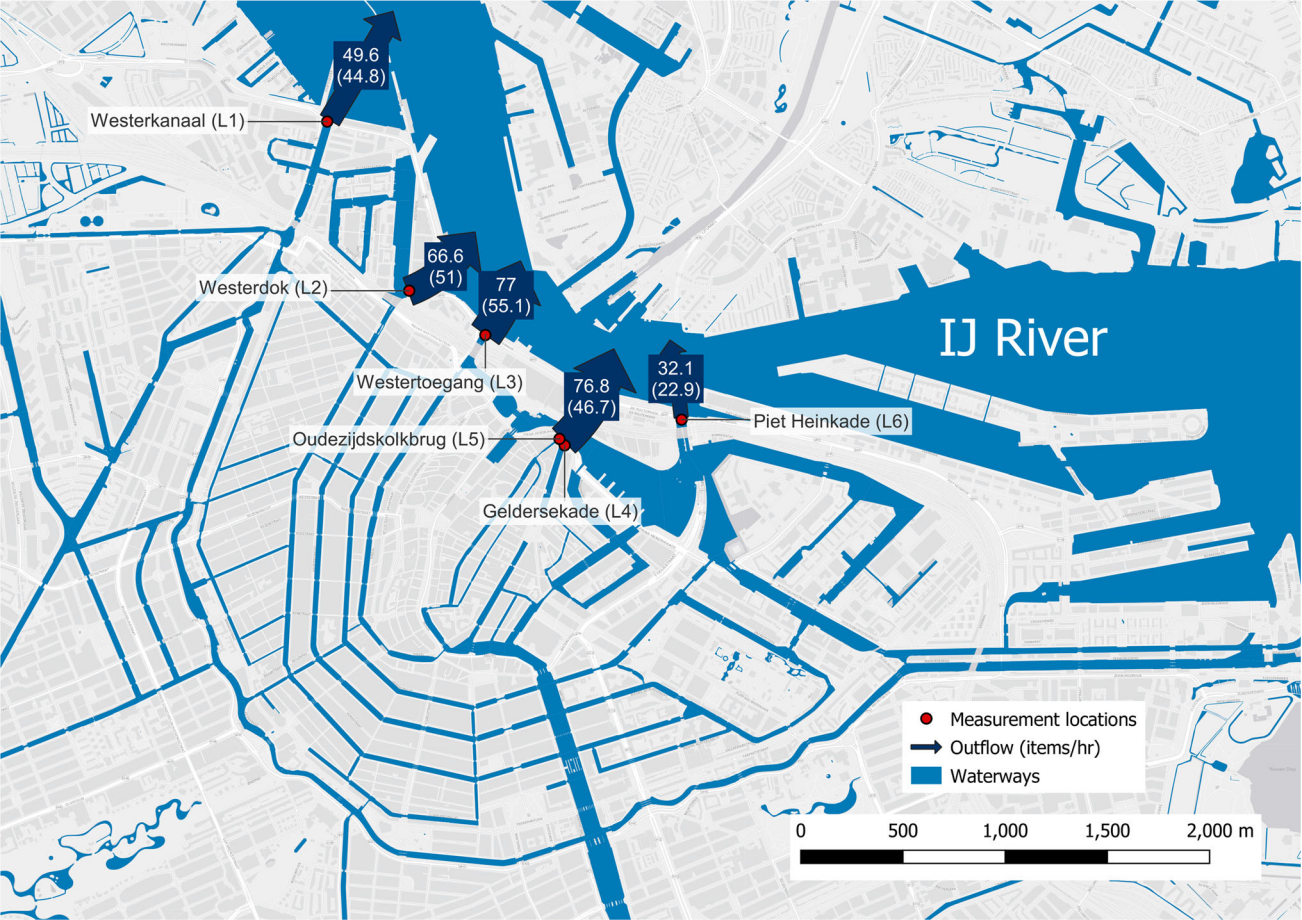
Litter fluxes for each outlet, expressed in items per hour. Geldersekade (L4) and Oudezijdskolkbrug (L5) are combined. Numbers in parentheses indicate plastic fluxes

To analyze local drivers impacting litter abundance, retention, and transport, litter fluxes were correlated to meteorological data. The meteorological variables were obtained from the Royal Netherlands Meteorological Institute (KNMI) data platform (https://dataplatform.knmi.nl/). Variables obtained from this data platform are as follows: daily sun hours, accumulated daily rainfall, rainfall duration (hours), average wind speed, maximum wind speed, and wind direction. These are derived using the Schiphol Airport weather station, located approximately 10 km  outside the Amsterdam city center. Information about tidal regimes (at IJmuiden, 20 km downstream of Amsterdam) was obtained from Rijkswaterstaat (Dutch Directorate-General for Public Works and Water Management, https://getij.rws.nl/). The MATLAB software was used to derive Pearson correlations between meteorological variables, tidal regimes, and observed floating litter fluxes. All data and scripts are included as supplementary material, as summarized in the data availability statement.

## Results and discussion

### Outlet emissions

A total of 1006 items were counted at six outlets over 13 months. Of these items, 735 (73%) were categorized as plastic according to the six different polymer categories. This percentage is comparable to Tramoy et al. (2022), in which 83% of anthropogenic items in urban water  systems were characterized as plastics. Aditionally, Luo et al. (2022) found plastic made up 70.3% of the total items found in littered mangroves close to Hongkong, which is also comparable to the 73% in our study. The largest amount of items observed were plastics in the “PO_Soft_” category (35%), followed by “Other” (27%) and “Multilayer” (23%). These high observations can be related to consumer products, such as shopping and grocery bags (PO_Soft_), cigarette butts (Other), and single-use food wrappers and packaging (Multilayer). The emissions of the other plastic categories “EPS” (6%), “PO_Hard_,” (5%), “PS” (2%), and “PET” (2%) are significantly smaller. van Emmerik et al. (2022) observed comparable shares of floating PO_Soft_ (39.5%), Multilayer (17.1%), EPS (7.7%), and PET (1.1%) in Dutch rivers, implying that these categories are possibly linked to emissions from urban water systems.

**Fig. 4:**
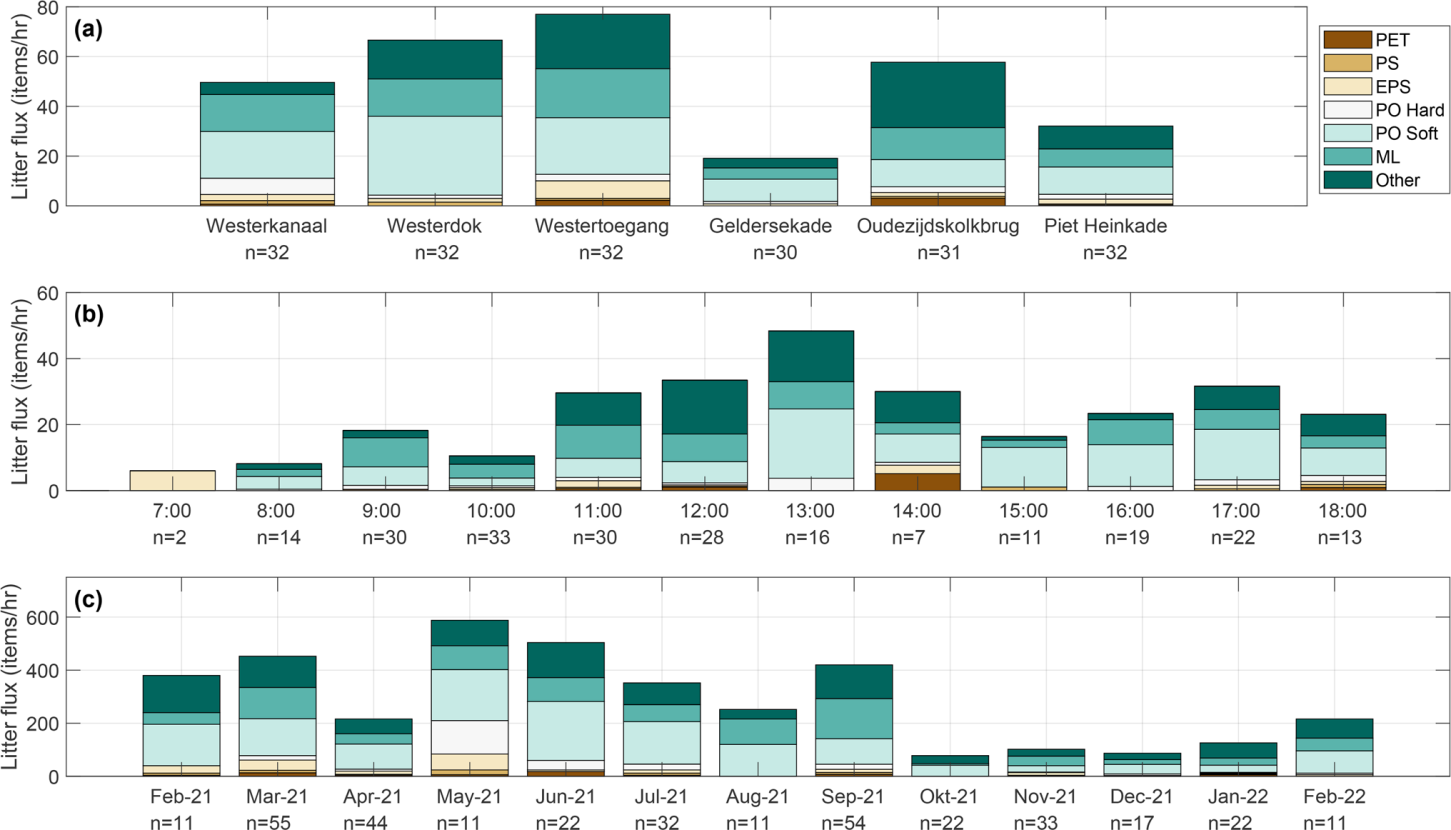
Overview of categorised litter fluxes, with **a** mean item fluxes per measurement location, **b** mean item fluxes per time of day, **c** mean item fluxes per month. For each flux, the number of individual measurements (*n*) is given

The item fluxes of the individual outlets are shown in Fig. 3. The total flux of all outlets combined was 302 items/h (221 plastic items/h), approximately 2.7 million items/year (1.9 million plastic items/year). These  flux values rank the Amsterdam canal system among the highest in comparison with 42 rivers in eleven European countries, of which the Danube River is the most polluted (3.0 million items/year) (González-Fernández et al. 2021). Relative errors for the measured item fluxes range between 15.5$-$27.9% items/h, and 17.4$-$30.1% plastic items/h. An overview per measurement location is included in the supplementary material. Interestingly, the estimated yearly flux of the Amsterdam system into the IJ river is similar to estimates of the Dutch Rhine (2.7$-$3.5 million items/year), IJssel (2.4$-$2.6 million items/year), and Meuse (2.3$-$3.8 million items/year) Rivers (van Emmerik et al. 2022), implying that urban water systems are major contributors to river plastic pollution. Converted to mass estimates based on mean category mass, approximately 39.5 metric tons of litter (19.5 metric tons of plastic) flows in the IJ river annually. The mass estimates based on median category mass are 2.7 metric tons of litter (2.6 metric tons of plastic). Large differences in mean and median weight statistics of plastic litter result in substantial uncertainties for litter emissions to the IJ river. Estimates based on mean litter weights can be an order of magnitude higher compared to estimates based on median litter weights. Yet, these mean and median estimates are well within the range of estimates for Dutch rivers by van Emmerik et al. (2022), further corroborating the major role of urban water systems in river plastic pollution.

### Spatiotemporal variation

Variations in litter type and abundance were observed between the different measurement locations (Fig. 4a), at hours of the day (Fig. 4b), and monthly variation (Fig. 4c). The highest litter emissions were observed at the Westertoegang bridge (L3, 77 items/h), at 13:00 (48.4 items/h), and in May 2021 (588 items/h). Possible causes for the variations in litter type and abundance include a range of explanations. Among others, these are (1) the presence of people and the intensity of human activity (McCormick and Hoellein 2016), (2) new policy measures to reduce litter emissions (Mihai et al. 2022), (3) the presence of traditional open-air markets (Cordova and Nurhati 2019), (4) COVID-19 regulations and impacts (Cordova et al. 2021), and (5) environmental drivers (Roebroek et al. 2021), which is discussed in the next subsection.

**Fig. 5:**
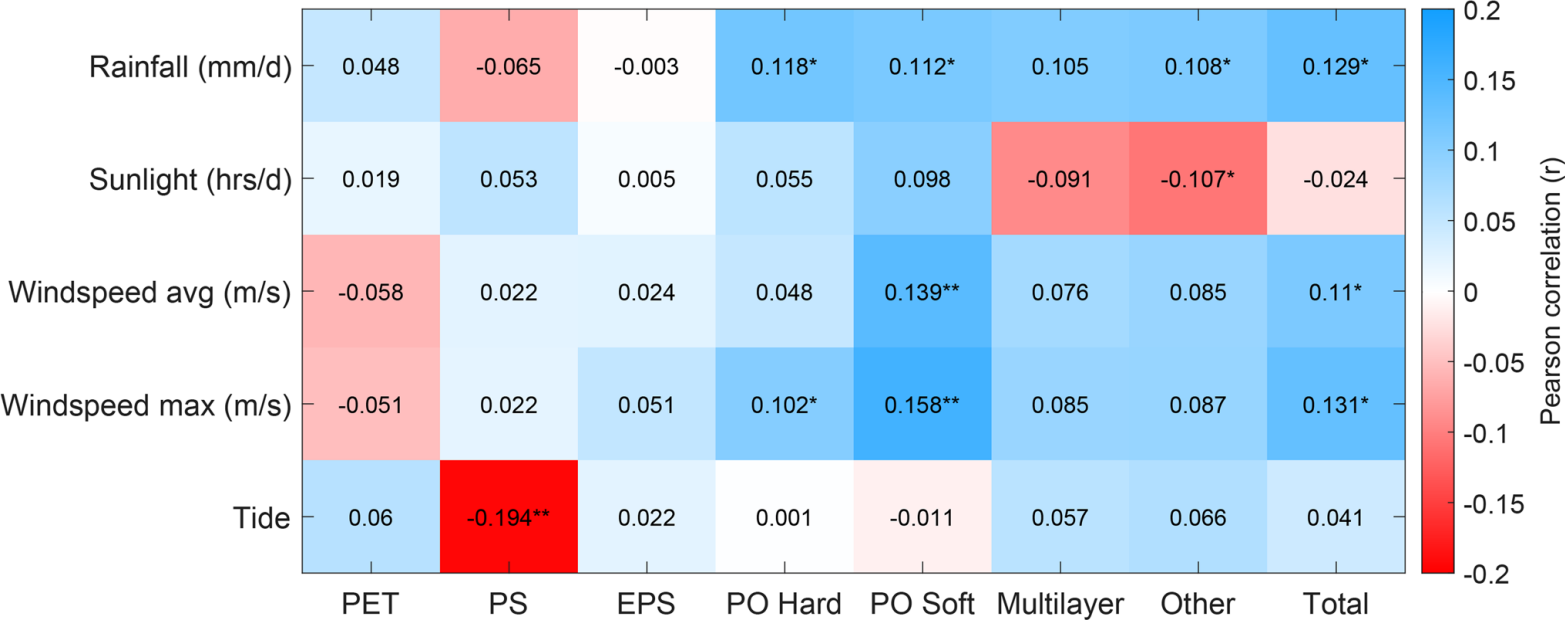
Pearson correlations between the litter categories used for visual counting and environmental factors. Stars indicate the level of significance: *p* $\le$ 0.05 (*), *p* $\le$ 0.01 (**)

Several variations in litter abundance depicted in Fig. 4 could be linked to these explanations. For instance, the number of people present and the intensity of human activities might cause immediate higher litter emissions to the channels (Ballatore et al. 2022). People can use areas in the vicinity of the canals as recreational areas (Kiessling et al. 2019), or dispose of waste illegally (Franz and Freitas 2012; Kiessling et al. 2021). Bridges discharging canals from the touristic city center discharge relatively more (66.6, 77, and 76.8 items/h) compared to bridges discharging areas with less human activity (49.6 and 32.1 items/h) (Fig. 3). Another factor that seemed to influence litter abundance is the behavior of street workers and maintenance personnel. For multiple measurements at various locations, it was observed that street workers used leaf blowers to purposely mobilize litter items from the sidewalks and streets into the canals. As depicted in Fig. 4b, low litter fluxes were observed early in the mornings (7:00–10:00), whereas this increased to peak around lunchtime (13:00). An increase in “PO_Soft_” and “Other” items throughout the day could be attributed to increased disposal of single-use consumer products. No measurements were done between 18:00 and 07:00, so fluxes during the night remain unknown. An example of new measures to reduce litter emissions is the introduction of a € 0.15 deposit on small PET bottles in the Netherlands in July 2021 Government of the Netherlands (2020). Yet, this does not result in a clear decrease in PET litter fluxes after this introduction (Fig. 4c). Even though these explanations could potentially lead to variation in litter abundance, additional monitoring close to actual sources, rather than at the outlets in the IJ River, is necessary.

### Environmental factors

Pearson correlations between environmental drivers and the observed litter fluxes are low, ranging from $-$0.19 to 0.16 (Fig. 5). During the measurements, wind gusts were observed to influence the mobilization and transport of floating litter at outlets, yet the correlation between the highest daily wind gusts (windspeed max (m/s)) and outflow of all item categories combined is low (0.13). Even though correlations between wind speed, rainfall, and observed item fluxes are low, the sign of the correlation is positive for most categories and comparable to the explanatory power and sign of environmental drivers found by Roebroek et al. (2021), who used multi-linear regression models to link various environmental factors with riverbank litter observations.

The other environmental factors (sunlight and tide) showed very weak correlations with observed item fluxes, with two exceptions (Tide-PS, and Sunlight-Other). It is possible that a non-trivial combination of factors determines the spatiotemporal variability of observed fluxes. In combination with the low correlation values of windspeed and rainfall, it is evident that the transport of floating litter in urban water systems is complex. The latter is corroborated by Roebroek et al. (2022), stating that multi-linear regression models only using environmental factors to explain plastic litter fluxes in rivers are unlikely to perform well. Anthropogenic activity, littering, and transport mechanisms should be included in such models, especially in urban areas where litter generation is concentrated (Treilles et al. 2021; Tramoy et al. 2022).

## Synthesis and outlook

### Complexity and drivers of litter transport

Anthropogenic litter pollution in urban water systems and subsequent transport to river systems is complex and dynamic. In this study, monthly visual counting measurements at six outlets of the Amsterdam urban water system resulted in an estimate of approximately 2.7 million items/year entering the IJ River. Even though this estimate is based on reliable observations, the current understanding of the impacts of potential drivers on the transport and retention of litter is limited. While environmental factors, such as (high intensity) rainfall events in urban areas could be drivers of litter transport to rivers (van Emmerik et al. 2022), it is argued that these factors on their own cannot fully explain the observed litter fluxes (Roebroek et al. 2022). The low correlation values between precipitation, sunlight, wind speed, tidal regimes, and observed item fluxes in our study confirm the latter. Therefore, understanding other drivers such as direct littering and stormwater overflow (Treilles et al. 2021), and intensity of anthropogenic activities (McCormick and Hoellein 2016; Cordova and Nurhati 2019) is key for future efforts. These efforts could focus on high-frequency monitoring at locations with a variety of indicators for anthropogenic activity: e.g., open-air markets, restaurants, city parks, public transport nodes, and other potential sources of emission.

### Local factors and mitigating measures

In addition, local factors and indirect drivers can influence litter abundance, retention, and transport. For instance, regulatory instruments to mitigate or prevent direct littering could promote sudden changes in anthropogenic behavior (Baxter et al. 2022). Other local factors include systems to collect litter, such as The Great Bubble Barrier structure in Amsterdam, or larger initiatives focusing on reducing outflow to marine ecosystems (e.g., Plastic Smart Cities https://plasticsmartcities.org/). Another factor includes targeted cleanups, such as the “Plastic Whale” initiative (https://plasticwhale.com/). This initiative collects floating litter from canals in Amsterdam, which is subsequently recycled to make furniture and fishing boats. While these instruments contribute to reducing litter abundance, they also influence estimates of litter transport from urban water systems to rivers and oceans. The latter is increasingly important for policymakers (van Emmerik et al. 2022), which emphasizes the need of including local factors in future estimations of litter transport. These efforts should also relate the abundance of (floating) litter with the presence of waste bins, open-air markets, restaurants, and other potential sources of emission. To these ends, it would be beneficial to expand the polymer-based categorization with waste sectors (i.e., “food,” “industry,” “housekeeping,” etc.). In summary, it is relevant to include both contributing factors (emissions) and mitigating factors (local cleanups and regulatory measures) of litter transport and couple these to waste sectors.

### Future research directions for advanced monitoring

Future research should explore additional monitoring techniques to quantify litter outflow. Since the relationship between floating plastics and total plastics in the water column is unclear (van Emmerik et al. 2022), the estimation of total outflow quantities based on just floating plastics could be inaccurate. Current technologies are either labor intensive and require heavy equipment (Oswalda et al. 2021; Blondel and Buschman 2022) or are based on rudimentary techniques, such as acoustic sonar (Broere et al. 2021; Flores et al. 2022). The Great Bubble Barrier could form the interface between these techniques, as it mobilizes litter suspended in the water column to the surface, where it is captured. Additional monitoring techniques involve camera systems on bridges or drones, either RGB (van Lieshout et al. 2020), multispectral (De Giglio et al. 2020; Biermann et al. 2020), or hyperspectral systems (Balsi et al. 2021; Tasseron et al. 2021; Cocking et al. 2022). Using these systems in Amsterdam could greatly improve the temporal resolution of datasets and reduce the labor-intensive visual counting from bridges. In addition, the strategic application of these systems contributes to the understanding of direct and indirect drivers, including tidal regimes and dynamic environmental conditions.

### Practical applications integration in communities

Finally, it is important to consider the practical applications of detailed monitoring techniques. At some point, well-defined types of litter with a clear origin, abundance, and transport mechanisms are determined. Communicating these results with local communities and municipalities could help to raise awareness and stimulate creative solutions to mitigate litter abundance and prevent emissions to urban water systems (Sandu et al. 2020). Various stakeholders in polluted areas such as restaurants, waste managers, and/or citizen/community-based initiatives could be involved in experiments to reduce litter emissions. Subsequent integration of the monitoring results, creative ideas, and experiments in urban living labs would provide an innovative inclusive environment for solutions to be smoothly and swiftly implemented (Steen and Van Bueren 2017).

## Conclusion

Urban water systems are estimated to be the main source of plastic pollution in rivers, seas, and oceans. The goal of this paper is to provide fundamental evidence for the latter, linking emission quantities and item categories to river plastic pollution. In this study, novel insights in assessing and quantifying the role of the Amsterdam urban water system as a source of river plastic pollution were delivered. Based on visual counting of floating litter from bridges, it is estimated that 2.7 million items enter the IJ river annually. This emission ranks the Amsterdam water system among Europe’s most polluted rivers observed to date.

Variations in litter type and abundance at various spatiotemporal scales include a range of possible explanations. Environmental drivers including wind, precipitation, sunlight, and tidal regimes lack strong correlations with observed item fluxes (*r* = $-$0.19–0.16). These results call for other factors such as the intensity of human activity, and the influence of point sources (street markets, restaurants) to be included in future correlation analyses.

Aditionally, the largest amounts of items were plastics in the “PO_Soft_” category (35%), which can be related to consumer products such as shopping and grocery bags. Yet, the categorization of litter items in future efforts should include more detailed item categories and include their waste sectors (i.e., “food,” “industry”). Communicating and integrating these results with local stakeholders in polluted areas could eventually provide an innovative environment for solutions to be efficiently implemented.

With this paper, we present a first one-year assessment of floating plastic emissions from the Amsterdam water system into the IJ River. We aimed to shed new light on plastic transport dynamics within urban water systems, and its contribution to river plastic pollution. Future research is needed to further disentangle the driving factors of the observed spatiotemporal variability.

## Data Availability

Data sheets and associated MATLAB files are available online at https://doi.org/10.4121/21369972.
